# High-resolution melt curve analysis: An approach for variant detection in the *TPO* gene of congenital hypothyroid patients in Bangladesh

**DOI:** 10.1371/journal.pone.0293570

**Published:** 2024-04-10

**Authors:** Mst. Noorjahan Begum, Rumana Mahtarin, Md Tarikul Islam, Nusrat Jahan Antora, Suprovath Kumar Sarker, Nusrat Sultana, Abu A. Sajib, Abul B. M. M. K. Islam, Hurjahan Banu, M. A. Hasanat, Kohinoor Jahan Shyamaly, Suraiya Begum, Tasnia Kawsar Konika, Shahinur Haque, Mizanul Hasan, Sadia Sultana, Taufiqur Rahman Bhuiyan, Kaiissar Mannoor, Firdausi Qadri, Sharif Akhteruzzaman

**Affiliations:** 1 Department of Genetic Engineering & Biotechnology, University of Dhaka, Dhaka, Bangladesh; 2 Institute for Developing Science and Health Initiatives (ideSHi), ECB Chattar, Mirpur, Dhaka, Bangladesh; 3 Infectious Diseases Division, Virology Laboratory, International Centre for Diarrhoeal Disease Research, Bangladesh, Mohakhali, Dhaka, Bangladesh; 4 Department of Endocrinology, Bangabandhu Sheikh Mujib Medical University (BSMMU), Shahbag, Dhaka, Bangladesh; 5 Department of Pediatrics, Bangabandhu Sheikh Mujib Medical University (BSMMU) Shahbag, Dhaka, Bangladesh; 6 Nuclear Medicine and Allied Sciences, Bangabandhu Sheikh Mujib Medical University (BSMMU), Shahbag, Dhaka, Bangladesh; 7 Scintigraphy Division, Bangabandhu Sheikh Mujib Medical University (BSMMU) Shahbag, Dhaka, Bangladesh; 8 Infectious Diseases Division, Mucosal Immunology and Vaccinology, International Centre for Diarrhoeal Disease Research, Bangladesh, Mohakhali, Dhaka, Bangladesh; University of Wisconsin-Madison, UNITED STATES

## Abstract

*TPO* (Thyroid Peroxidase) is known to be one of the major genes involved in congenital hypothyroid patients with thyroid dyshormonogenesis. The present study aims to validate high-resolution melting (HRM) curve analysis as a substitute method for Sanger sequencing, focusing on the frequently observed non-synonymous mutations c.1117G>T, c.1193G>C, and c.2173A>C in the *TPO* gene in patients from Bangladesh. We enrolled 36 confirmed cases of congenital hypothyroid patients with dyshormonogenesis to establish the HRM method. Blood specimens were collected, and DNA was extracted followed by PCR and Sanger sequencing. Among the 36 specimens, 20 were pre-sequenced, and variants were characterized through Sanger sequencing. Following pre-sequencing, the 20 pre-sequenced specimens underwent real-time PCR-HRM curve analysis to determine the proper HRM condition for separating the three variations from the wild-type state into heterozygous and homozygous states. Furthermore, 16 unknown specimens were subjected to HRM analysis to validate the method. This method demonstrated a sensitivity and specificity of 100 percent in accurately discerning wild-type alleles from both homozygous and heterozygous states of c.1117G>T (23/36; 63.8%), c.1193G>C (30/36; 83.3%), and c.2173A>C (23/36; 63.8%) variants frequently encountered among 36 Bangladeshi patients. The HRM data was found to be similar to the sequencing result, thus confirming the validity of the HRM approach for *TPO* gene variant detection. In conclusion, HRM-based molecular technique targeting variants c.1117G>T, c.1193G>C, and c.2173A>C could be used as a high throughput, rapid, reliable, and cost-effective screening approach for the detection of all common mutations in *TPO* gene in Bangladeshi patients with dyshormonogenesis.

## 1. Introduction

Congenital hypothyroidism (CH) is the endocrine disorder in which thyroid hormone deficiency occurs at birth and is the most common preventable cause of mental retardation [1–3]. This condition can lead to different clinical complications such as irreversible brain damage, delayed developmental milestones, lethargy, and slowdown of the body’s overall metabolism of the patients if untreated. Early detection and initiation of treatment can reverse such complications [4]. The incidence of CH is more than twice (1 in 1300) the global incidence rate (1 in 3000–4000) in Bangladesh [5–9]. However, large-scale studies on congenital hypothyroidism are inadequate in Bangladesh. In CH condition, 11 genes, including in thyroid gland development such as *TSHR* (thyroid stimulating hormone receptor, **+** 603372) and three transcription factors- *TTF-1* (*600635), *TTF-2* (*602617), and *PAX8* (paired box-8, transcription factor, *167415) and thyroid hormone biosynthesis such as *NIS* (sodium iodine symporter, SLC5A5, *601843), *PDS* (Pendrin or SLC26A4, *605646), *TPO* (thyroid peroxidase, *606765), *TG* (thyroglobulin, *188450), *IYD* (iodotyrosine deiodinase, DEHAL1, *612025), *DUOX2* (dual oxidase 2, *606759), and *DUOXA2* (dual oxidase maturation factor 2, *612772), have been documented [10–13].

A defect in thyroid gland development due to mutations in both gene alleles of the pathway is known as thyroid dysgenesis. In contrast, a defect in thyroid hormone biosynthesis due to mutations in both alleles of a gene of the pathway is called thyroid dyshormonogenesis (TDH). *TPO* gene variants are a significant contributor to TDH [14,15]. Since thyroid hormones are iodinated, TPO catalyzes the iodination steps, and mutations in the *TPO* gene may cause either total iodide organification defect (TIOD) or partial iodine organification defect (PIOD). Different countries conducted several studies on screening and identification of variants in the *TPO* gene causing TIOD and PIOD [16–21]. We found no additional aetiology in our prior work, which examined whether only genetic reasons could explain for all CH patients with dyshormonogenesis in hospital settings in Bangladesh [11]. Since CH is easily treatable, it is important to investigate the aetiology, which would help to determine how long the patients need hormone replacement therapy. The CH patients with genetic aetiology need lifelong hormone therapy [22]. Sometimes, neonatal CH screening using biochemical tests becomes difficult due to the presence of maternal TSH in the specimens of neonates, and this problem can be overcome by genetic screening at an early age [22]. There is limited data on investigating the genetic causes of CH in Bangladesh. However, we have performed several genetic studies and found variants in patients with thyroid dyshormonogensis [11,12] and thyroid dysgenesis [13]. When investigating variants in *TPO* gene in dsyshormonogenesis, a total of four variants, namely, c.1117G>T (p.Ala373Ser), c.1193G>C (p.Ser398Thr), c.2145C>T (p.Pro715Pro), and c.2173A>C (p.Thr725Pro) were identified in the study participants [11]. Also, we identified two nonsynonymous variants, c.1523C>T (p.Ser508Leu) and c.2181G>C (p.Glu727Asp) in the exon 10 of the *TSHR* gene in 21 patients with dysgenesis by sequencing-based analysis [13].

High-resolution melting (HRM) curve analysis is one of the molecular tests, which is a high throughput real-time PCR technique based on the melting properties of double-stranded DNA. HRM can differentiate genetic variations such as homozygous or heterozygous states for specific mutations compared to the wild-type state in various genetic diseases, including autosomal recessive, autosomal dominant, and X-linked recessive disorders [23–26]. However, worldwide, there is very limited data on CH based on HRM, one study detected variants in *DNAJC17*gene in thyroid dysgenesis by HRM method [27].

This study aimed to validate HRM curve analysis as a screening tool for congenital hypothyroidism (CH) in Bangladeshi patients, focusing on common genetic variations. By providing a faster, more affordable, and dependable method of mutation detection, it seeks to enhance treatment selection and address the lack of attention to newborn screening in Bangladesh.

## 2. Materials and methods

### 2.1. Study participants enrolment

We recruited 36 confirmed cases of congenital hypothyroid children with dyshormonogenesis who were undergoing follow-up treatment at National Institute of Nuclear Medicine and Allied Sciences (NINMAS) and the Department of Endocrinology, Bangabandhu Sheikh Mujib Medical University (BSMMU), Dhaka, Bangladesh. Our study physicians conducted thyroid scans and performed TSH, T3, T4 and Anti-TPO tests on the participants during enrolment. Their previous record showed that most of them were late diagnosed. Among them, who were confirmed at birth, initially, their blood TSH (Thyroid Stimulating Hormone) levels were assessed via a heel prick. If blood TSH levels were ≥20 mU/L, further analysis of peripheral blood FT4 (Free T4) was conducted to assess hypothyroidism risk. A diagnosis of congenital hypothyroidism was considered if initial TSH levels were >30 mU/L and initial T4 levels were below the 10th percentile. Moreover, clinical complications such as prolonged jaundice at birth, dry skin, constipation, delayed developmental milestones, lethargy, umbilical hernia, weight gain, open fontanelle, protruded tongue, hoarse voice, and a puffy face were also taken into account by the physicians for diagnosing CH. All the study individuals were undergoing treatment with Levothyroxine (LT4) during the study period and adjusted the drug dose by regular testing TSH and T4 level every 2 months.

### 2.2. Ethics approval and consent to participate

This study was approved by the Ethical Review Board for Human Studies of BSMMU and the Human Participants Committee, University of Dhaka (CP-4029). Blood specimens were collected from the participants with informed written consent from their parents or guardians by taking into account the regulations of WHO, international guidelines for biomedical research as laid down by the Declaration of Helsinki in relation to biomedical research involving human participants [28].

### 2.3. Laboratory investigation

#### 2.3.1. DNA isolation, PCR amplification, and Sanger sequencing

Whole blood specimens (3 mL) were collected, and genomic DNA was isolated using the QIAGEN FlexiGene^®^ DNA Kit, followed by PCR and Sequencing. During sequencing data analysis, *TPO* gene reference sequence (Accession number; NC_000002.12) was retrieved from the NCBI database [11]. Later, 20 pre-sequenced specimens were subjected to HRM, and then 16 unknowns were tested.

#### 2.3.2. Method setup, optimization, and validation of High-resolution melt curve analysis

For analysis of *TPO* gene variants by HRM method, we targeted three nonsynonymous variants in the *TPO* gene identified by Sanger sequencing. For this purpose, we designed three sets of primers for detecting nonsynonymous variants c.1117G>T and c.1193G>C in exon 8 and c.2173A>C in exon 12. The primer sequences are listed in S1 Table. First of all, those mentioned above, 20 pre-sequenced specimens with known variants were used as reference samples to set up and optimize the HRM method. Homozygous, heterozygous, and wild-type specimens for the specific variants were subjected to HRM curve analysis. Finally, 16 unknown samples were run to validate the method. These 16 samples were further tested by Sanger sequencing to confirm the variant and validated the HRM approach.

To amplify the target sequence, a master mix was prepared following the protocol provided with the Precision Melt Supermix kit (Bio-Rad) (S2 Table). Moreover, for detection of c.1193G>C variant by HRM, 8mM MgCl_2_ was added to the reaction mixture, and the reaction volume was adjusted accordingly. The cyclic condition was divided into two steps, namely real-time PCR amplification followed by melt curve analysis using a single program (S3 Table). During melting, temperature increment of 0.1°C per 5 s was for c.1117G>T and c.2173A>C variants. And notably, an increment of 0.2°C per 5 s was used for c.1193G>C variant. The real-time PCR-based HRM was performed on a CFX96 Touch^™^ Real-Time PCR machine (Bio-Rad). After completion of the real-time PCR-HRM, the data were analyzed using Precision Melt Analysis^™^ Software (BioRad). The melt curve shape sensitivity for cluster detection was set to 100%. The difference in the Tm threshold for the cluster detection was set to 0.1 to 0.2, and the normalized and temperature-shifted views were used for analysis. The results of pre-sequenced samples were compared with HRM analysis, and an additional 16 unknown samples were subjected to validate the HRM method using the same procedure that was followed for the pre-sequenced known samples. Finally, the normalized melt curve and the difference curves for both wild-type and mutant specimens (homozygous and heterozygous) were visualized and analyzed to detect the variants in the *TPO* gene.

## 3. Results

### 3.1. Metadata of the study participants

Among 36 hypothyroid participants, 21 (58.33%) were males, and 15 (41.67%) were females. The average age of the participants was 7.97±4.29 (years) mean± SD, and the BMI was 17.0±4.4 (Kg/m^2^) mean± SD. All the patients received thyroid hormone replacement therapy on daily basis and LT4 dosages were adapted based on age, sex and BMI of the patients. Before initiation of hormone replacement therapy, the mean serum TSH level of the patients was 66.07±57.38 mIU/L, while the baseline TSH level was 3.61±4.4 mIU/L after LT4 therapy. All the patients enrolled in the study had a normal serum free T4 (FT4) level (17.68±5.09 pmol/L) after LT4 therapy. However, the study could not compare the FT4 levels of pre-treatment period with that of posttreatment period because the former was not recorded for majority of the patients.

Among the 36 Bangladeshi patients, the presence of the c.1117G>T variant was observed in 23 individuals (63.8%), while the c.1193G>C variant was detected in 30 patients (83.3%). Additionally, the c.2173A>C variant was identified in 23 patients (63.8%). These variants were observed across various genotypic states, including wild-type, homozygous, and heterozygous configurations.

### 3.2. Screening of c.1117G>T variant using HRM analysis

**Fig 1:**
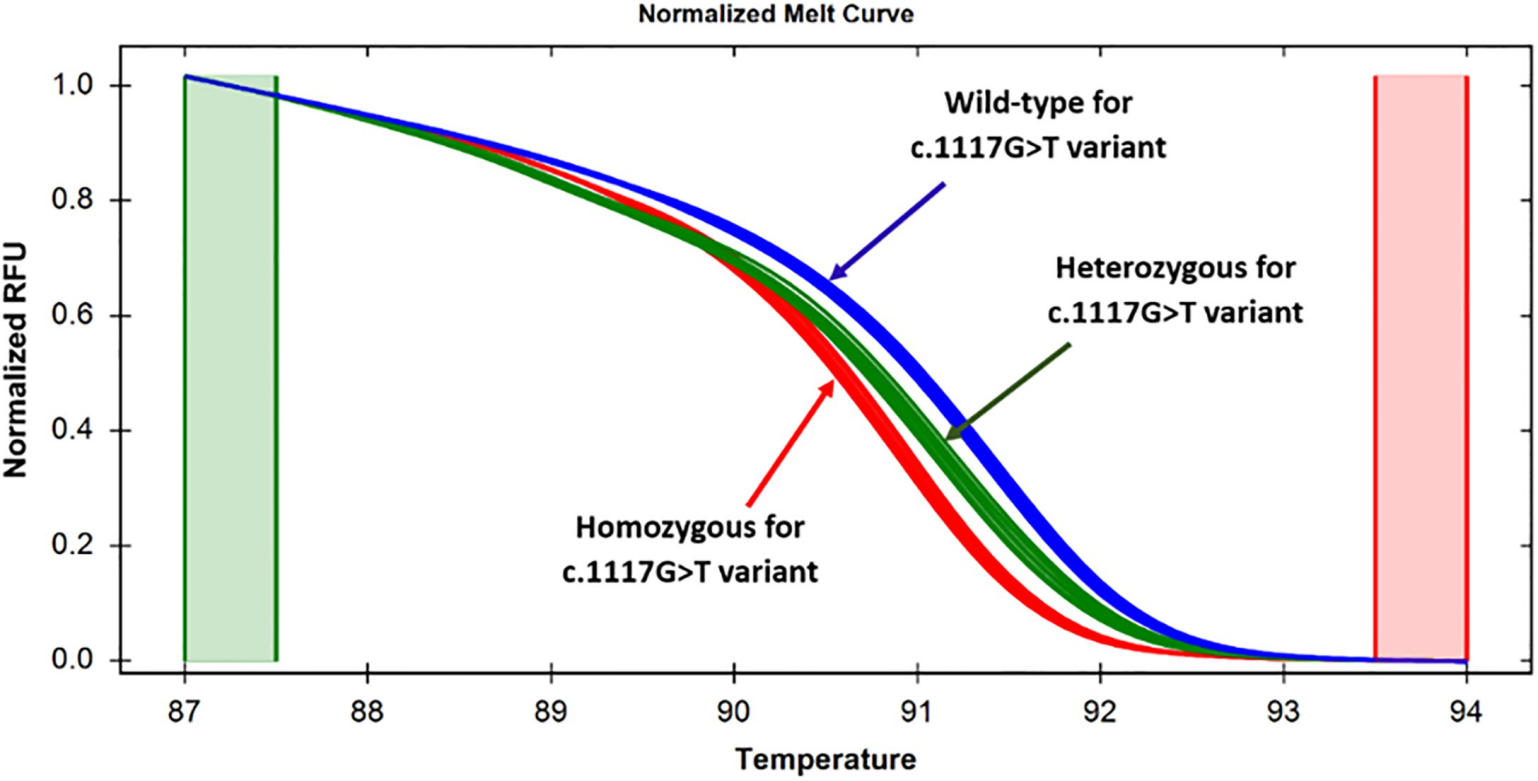
Normalized melt curves for the specimens targeting the c.1117G>T variant in exon 8. Normalized melt curves were showing that the specimens with homozygous and heterozygous states were clearly distinguishable from the wild-type specimens, as manifested by the difference in relative fluorescence unit.

**Fig 2:**
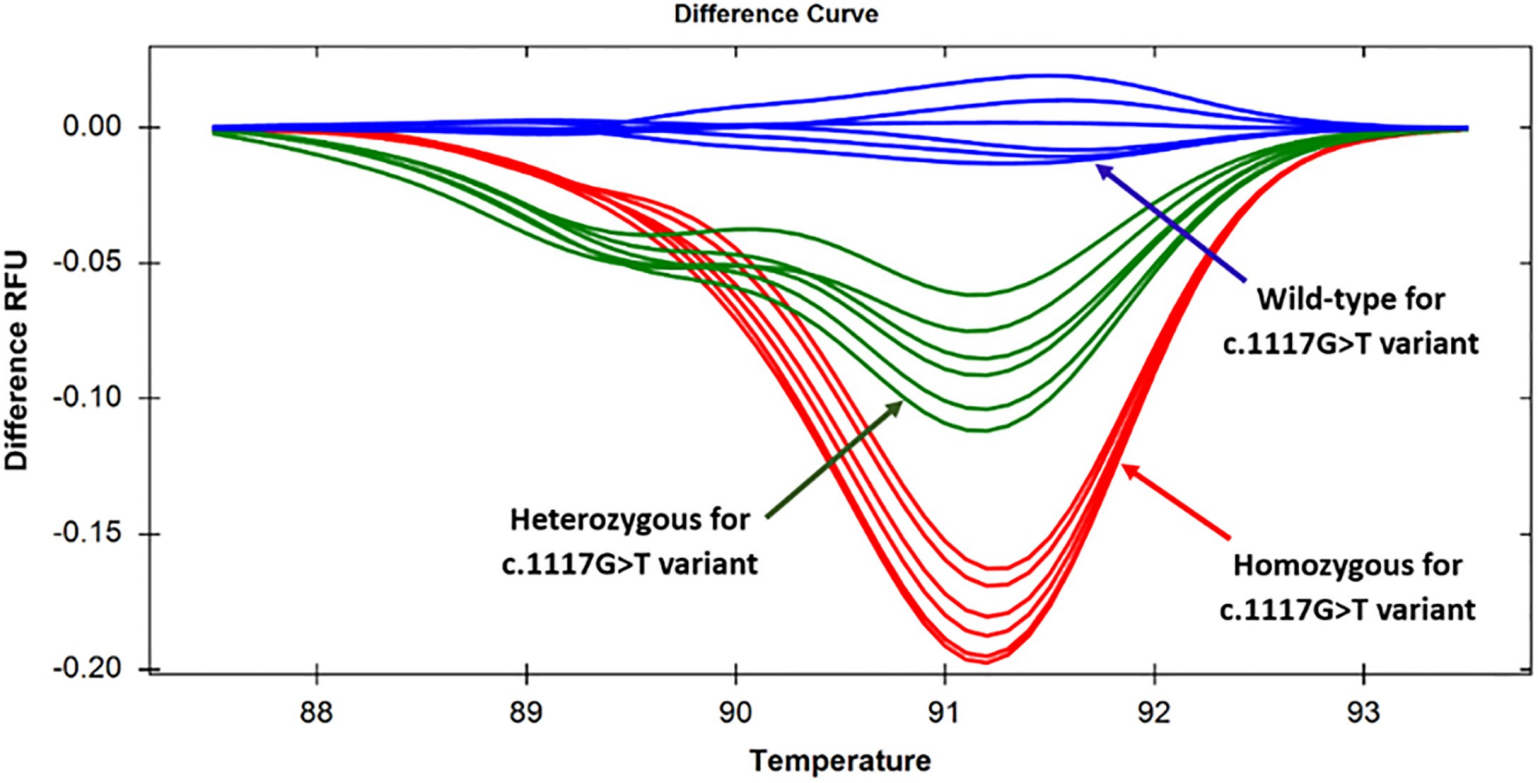
Difference curves generated by specimens targeting the c.1117G>T variant in exon 8. Discernable changes in three difference curves were showing that the specimens with homozygous and heterozygous states were clearly distinguishable from the wild-type allele, as manifested by the difference in relative fluorescence unit.

To establish a rapid HRM-based screening approach targeting the c.1117G>T variant, a pair of primers (TPO_G1117T_Ex8) was designed that flanked the c.1117G>T variant. Then, 20 pre-sequenced samples (S4 Table) were subjected to real-time PCR followed by HRM analysis. Similar to the normalized melting curve (Fig 1), the temperature-shifted difference curve could generate distinctive melting patterns for the wild-type, homozygous and heterozygous specimens (Fig 2). Among 20 known samples, 3 (15%) homozygous, 10 (50%) heterozygous and 7 (35%) wild-type states were differentiated for this variant.

The reliability of the method was further validated by analyzing 16 unknown samples with dyshormonogenesis. At first, these samples were tested by HRM, and then Sanger sequencing was done to check the sensitivity and specificity of the method (S5 Table). Among 16 samples a total of 5 (31.25%) specimens had c.1117G>T variant in homozygous states, 5 (31.25%) specimens had heterozygous states, and the remaining 6 (37.5%) had the wild-type alleles. The HRM result was consistent with the sequencing data, implying that sensitivity and specificity for detecting the c.1117G>T variant was 100% for both homozygous and heterozygous alleles. In summary, among the 36 patients, 8 (22.2%) exhibited a homozygous state, 15 (41.7%) displayed a heterozygous state, and 13 (36.1%) were classified as wild-type for the c.1117G>T variant.

### 3.3. Screening of c.1193G>C variant using HRM analysis

**Fig 3:**
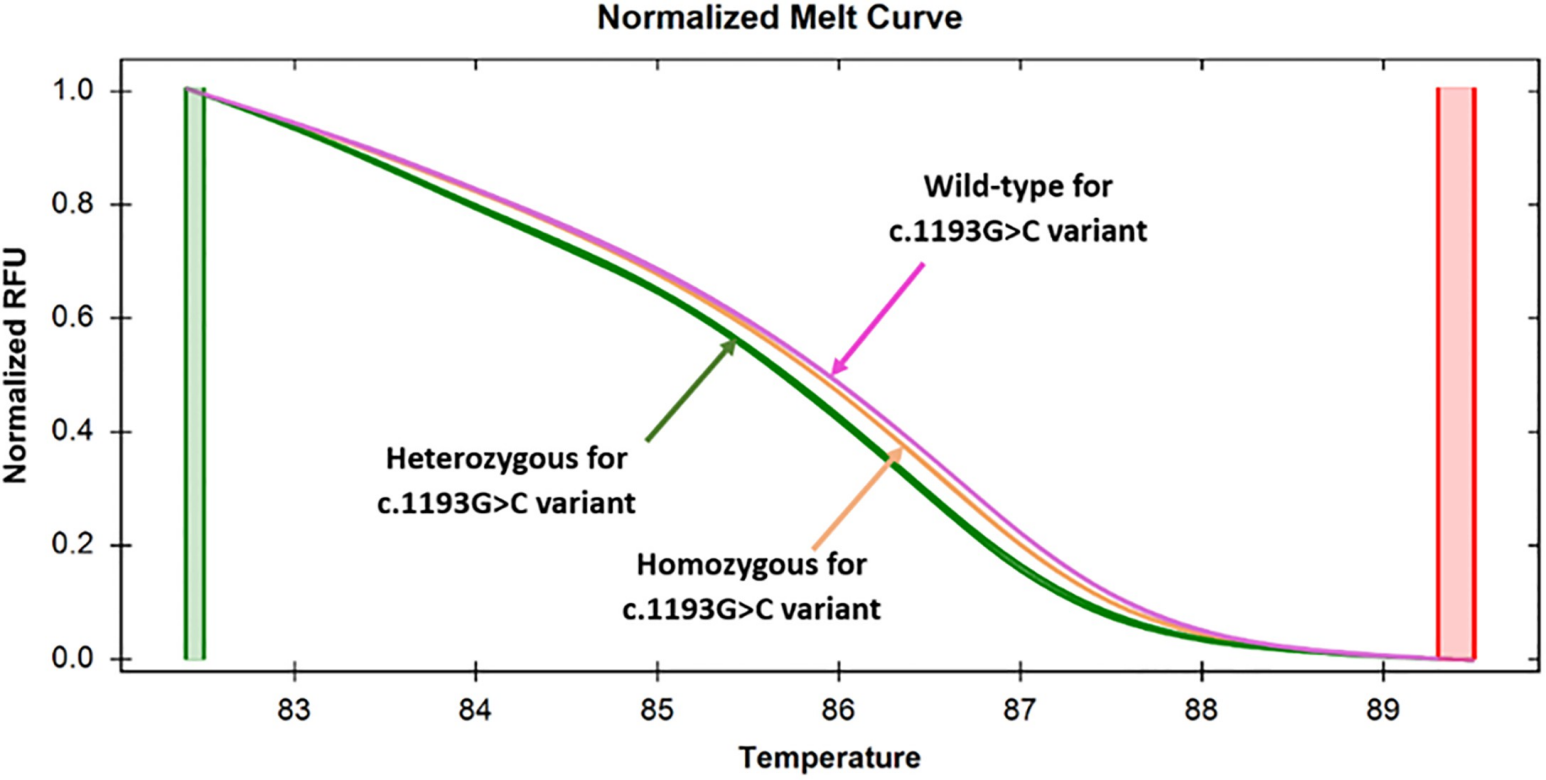
Normalized melt curves generated by specimens targeting the c.1193G>C variant in exon 8. Discernable changes in normalized melt curves were showing that the specimens with homozygous (orange color) and heterozygous (green color) states were clearly distinguishable from the wild-type (purple color) alleles, as manifested by the difference in relative fluorescence unit.

**Fig 4:**
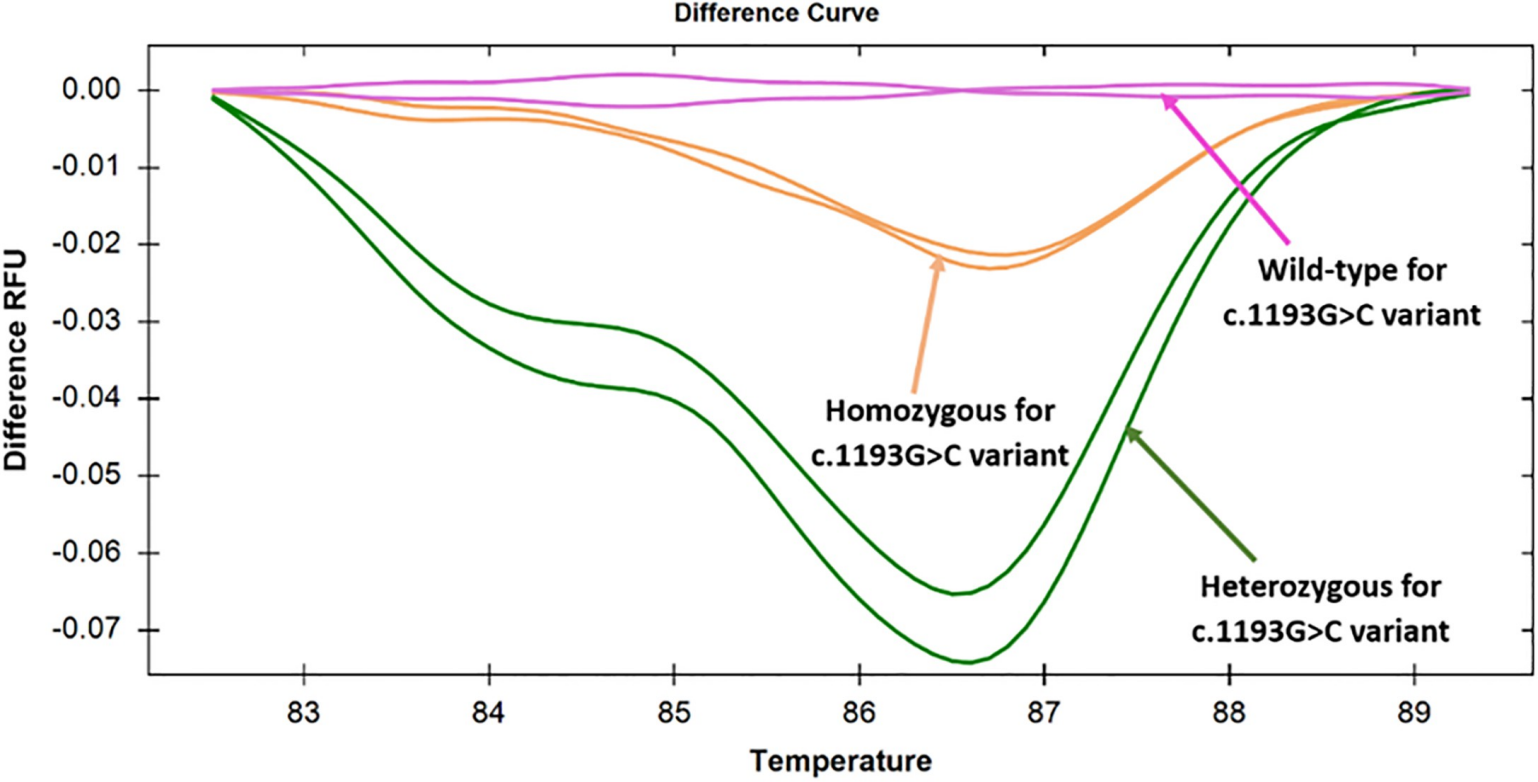
Difference curves for specimens targeting the c.1193G>C variant in exon 8. Discernable changes in difference curves were showing that the specimens with homozygous and heterozygous states were clearly distinguishable from the wild-type states, as manifested by the difference in relative fluorescence unit.

The second set of primers, namely TPO_G1193C_Ex8, was used for the analysis of the c.1193G>C variant by the HRM approach. When 20 pre-sequenced samples were subjected to HRM analysis, three different clusters were observed in the melt curve analysis. One of the clusters corresponded to the heterozygous samples; the other two were for the homozygous samples and for the wild-type samples (Fig 3). However, in the difference curve analysis, three different clusters were clearly observed for homozygous, heterozygous, and wild-type variants of c.1193G>C (Fig 4). Among 20, 11(55%) homozygous, 7 (35%) heterozygous, 2 (10%) wild-type states were differentiated for this variant.

A similar observation was observed with 16 unknown samples that were subjected to HRM analysis. 4 (25%) out of 16 samples came out as heterozygous by HRM analysis, and this result was consistent with the sequencing data. However, 8 (50%) homozygous and 4 (25%) wild-type samples formed different clusters in this case. This observation implies that both heterozygous and homozygous states for c.1193G>C variant could be detected with 100% sensitivity and specificity. Among the 36 patients, 19 (52.8%) exhibited homozygosity, 11(30.5%) displayed heterozygosity, and 6 (16.7%) were characterized as wild-type for the specified variant.

### 3.4. Screening of c.2173A>C variant using HRM analysis

**Fig 5:**
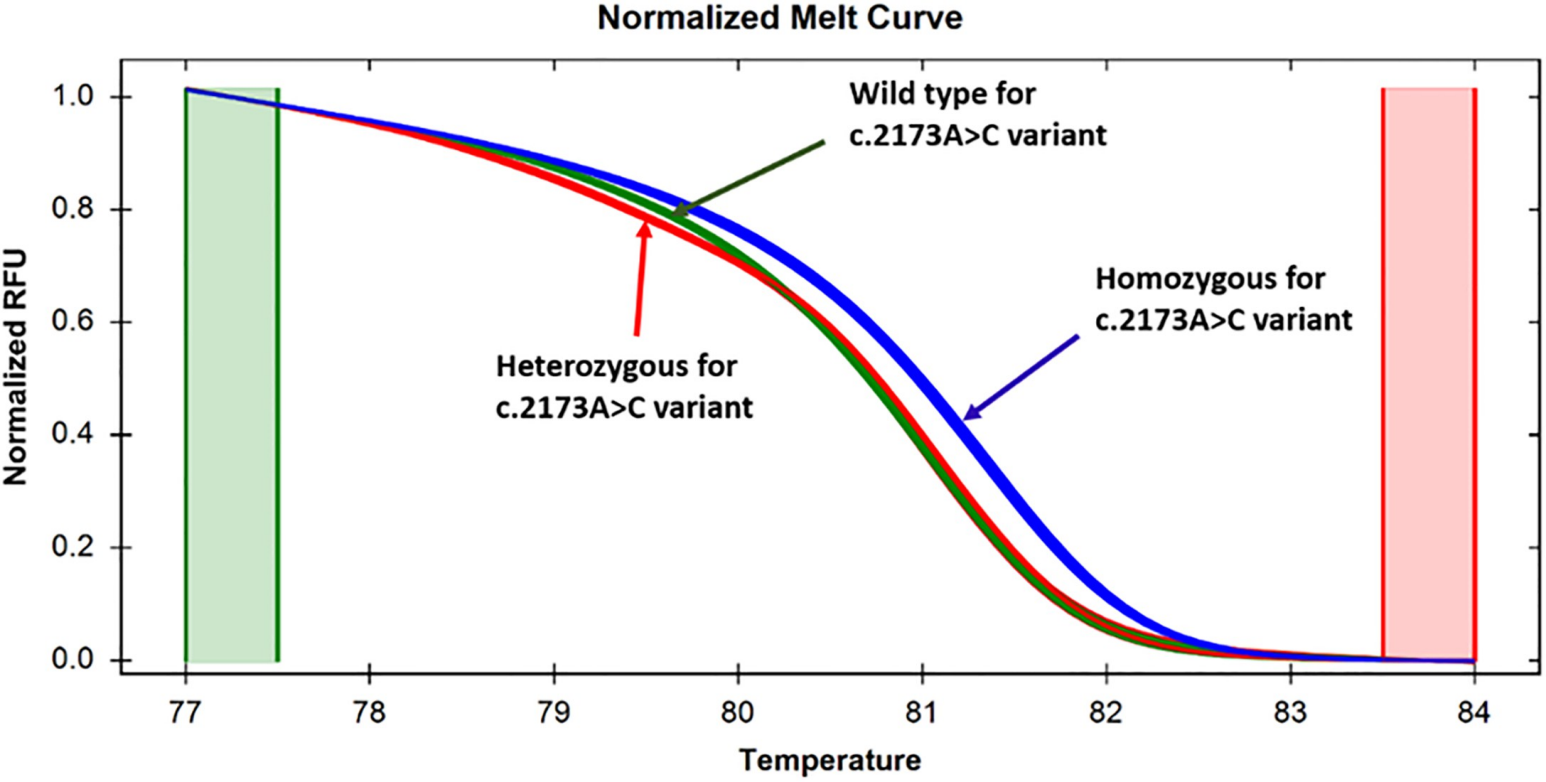
Normalized melt curves for specimens targeting the c.2173A>C variant in exon 12. Discernable changes in normalized melt curves were showing that the specimens with homozygous and heterozygous states were clearly distinguishable from the wild-type alleles, as manifested by the difference in relative fluorescence unit.

**Fig 6:**
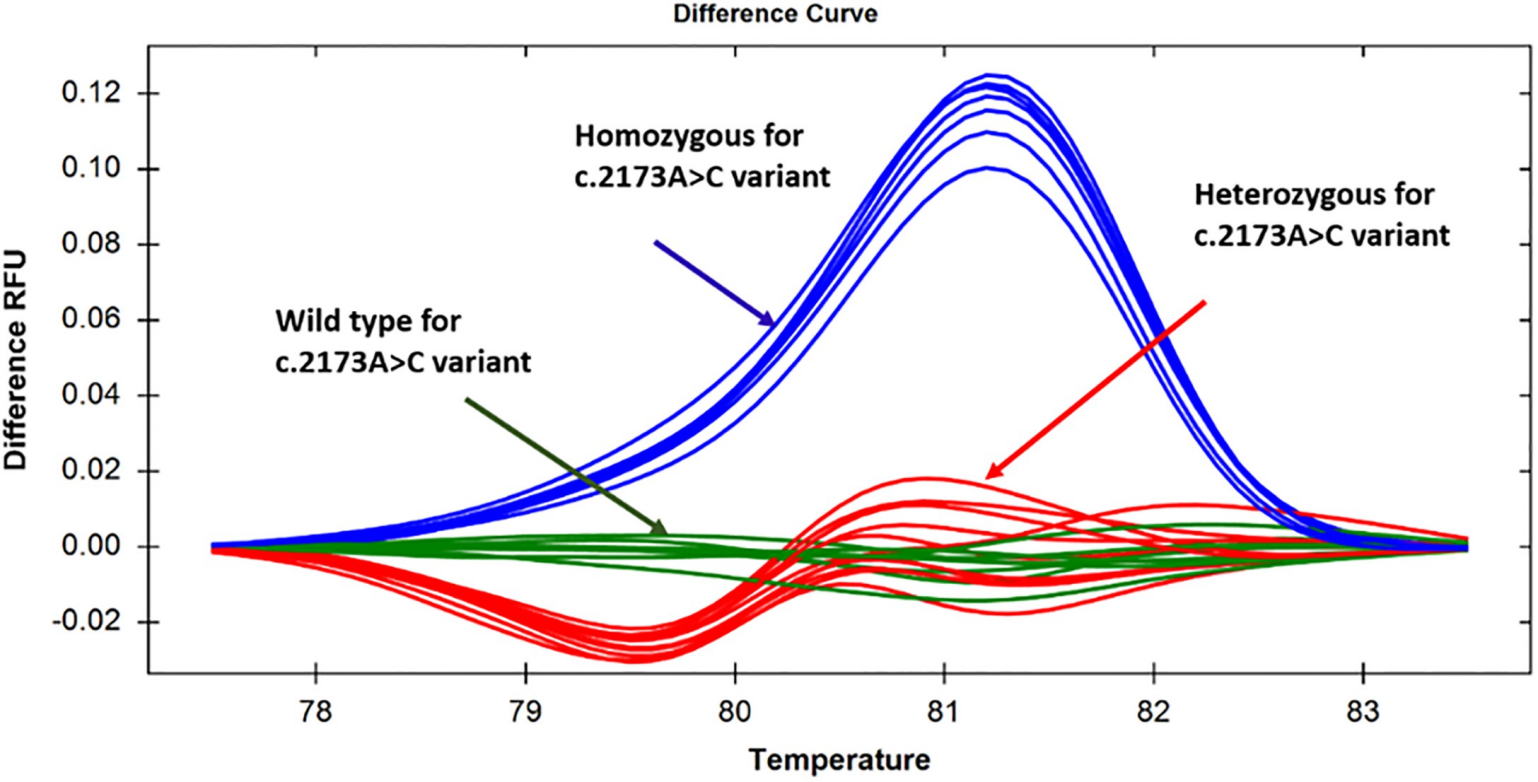
Differential curves for specimens targeting the c.2173A>C variant in exon 12. Discernable changes in difference curves were showing specimens with homozygous and heterozygous states were clearly distinguishable from the wild-type, as manifested by the difference in relative fluorescence unit.

The third set of primers, namely TPO_A2173C_Ex12, was used to analyze another *TPO* gene variant designated as c.2173A>C. The pre-sequenced wild-type, homozygous c.2173A>C, and heterozygous c.2173A>C specimens were subjected to HRM analysis. The wild-type, homozygous and heterozygous variants formed distinct clusters (Figs 5 and 6). The homozygous c.2173A>C substitution resulted in an increase in melting temperature, and thus, the specimens with homozygous c.2173A>C variant had higher fluorescence intensity than that with the wild-type allele during melting, as manifested by the relative fluorescence unit (Fig 5). On the other hand, although the specimens with the heterozygous c.2173A>C variant followed a melting pattern with lower fluorescence intensity initially compared to the wild-type, and the melting curve patterns almost overlapped with each other in a later stage (upper panel of Fig 5). Thus, homozygous c.2173A>C, heterozygous c.2173A>C, and the wild-type alleles were discernable from each other. Similar to the normalized melting curve, the difference curve analysis could also distinguish different states involving the c.2173A>C variant (Fig 6). Among the 20 patients examined, we identified 5 (25%) instances of homozygosity, 7 (35%) instances of heterozygosity, and 8 (40%) instances of the wild-type state for the variant in question.

HRM analysis of 16 unknown samples targeting the c.2173A>C variant was also 100% sensitive and specific. The HRM approach showed that 5 (31.25%) out of 16 unknown samples were wild-type, 6 (37.5%) were homozygous, and the rest 5 (31.25%) were heterozygous. Sequencing of those 16 unknown samples revealed that the PCR HRM-based result was consistent with the sequencing result. Within the group of 36 patients, 11 (30.5%) were identified as homozygous, 12 (33.3%) as heterozygous, and 13 (36.1%) as wild-type for the specified variant.

## 4. Discussion

Congenital hypothyroidism (CH) is the most common cause of developmental delay in children [29]. If early detection of CH is performed and treatment is initiated within 28 days of birth, clinical complications can be reversed by treatment with Levothyroxine, which is very easy to administer and affordable. Among the reported genes that are responsible for all CH cases with genetic aetiology, thyroid peroxidase (*TPO*) is one of the major genes for thyroid dyshomonogenesis, and its variants are inherited in an autosomal recessive manner to cause the disease [16,30,31]. TPO enzyme catalyzes the iodine oxidation process in the thyroid hormone synthesis pathway [32]. To date, approximately 60 mutations in the *TPO* gene have been reported in a total of 17 exons in the *TPO* gene [17,33–35]. Global publications on the *TPO* gene in hypothyroid patients demonstrated that most of the mutations were confined between exon 7 and exon 14, and very few mutations had been identified outside this region [34,35]. The identified nonsynonymous variants had previously been reported to be pathogenic or disease-causing mutations [15,21].

A previous genetic study investigated that mutation c.1117G>T and c.2173A>C showed a non-enzymatic reaction rate, and mutation c.1193G>C showed a slightly reduced enzymatic reaction rate compared to the wild-type TPO protein [36]. Our previous study identified four common variants in the hotspot region from exon 8 to exon 12 in the *TPO* gene and studied their effect on the 3D structure of the TPO protein [11]. Since we found these common variants in Bangladeshi patients, we aimed to establish an alternative method of Sanger sequencing to screen the patients. In Bangladesh, there is very little information about newborn screening and the genetic aetiology of CH. Bangladesh lies in a low- and middle-income country with no normal practice of newborn screening. Since, we enrolled all the congenital hypothyroid children in a stage of developmental delay. It is important to differentiate the patients whether they carry genomic mutations or acquire reason for iodine deficiency for congenital hypothyroidism.

High-resolution melting (HRM) methodology represents a significant advancement in variant detection over the years. The HRM method has been established for the detection of variants of the beta-globin gene in thalassemia patients and G6PD deficiency in Bangladesh [24,37]. Also, HRM approach brings profuse advantages over other existing methods for screening variants due to its simplicity, accuracy, and cost-effectiveness [24].

There are some screening methods for the diagnosis of CH, such as measurement of serum/blood TSH, T3, and T4. However, these approaches can only confirm the CH cases but not the actual aetiology. That is, the conventional screening method for CH cannot say whether it is acquired or genetic. If the actual aetiology is known, the duration of treatment can be defined based on the causes. If it is due to a genetic cause, the patients could be enrolled for Levothyroxine treatment for their whole life. On the other hand, treatment should be continued for the first three years of life for an acquired cause [22]. So, the treatment strategy will be different for CH cases with genetic aetiology and other reasons for CH with dyshormonogenesis. If the genetic basis of CH is defined in the country, carrier screening is possible to target the underlying genetic cause. If the parents are found to be carriers of CH involving the *TPO* gene, their children or newborns could be screened, and appropriate measures can be taken, such as early initiation of treatment, which would help to prevent mental retardation. Late diagnosis of CH is common in our country, and an initial pilot study suggested that late-diagnosed hypothyroid children had clinical complications even under Levothyroxine treatment in Bangladesh. So newborn screening should be a common practice for early CH diagnosis to prevent mental retardation due to late diagnosis.

The present study aimed to validate HRM method for the targeted variants (c.1117G>T, c.1193G>C, and c.2173A>C) commonly found in Bangladeshi CH patients. Among the 36 Bangladeshi patients, the presence of the c.1117G>T variant was observed in 23 individuals (63.8%), while the c.1193G>C variant was detected in 30 patients (83.3%). Additionally, the c.2173A>C variant was identified in 23 patients (63.8%). These variants were observed across various genotypic states, including wild-type, homozygous, and heterozygous configurations. To optimize and validate the method, we designed primers covering the mutational hotspot, keeping the product size between 65–101 base pairs, which fulfilled the requirement of the HRM strategy [38]. To establish HRM, the samples with heterozygous, homozygous, and wild-type alleles were subjected to an experiment. For the first set of primers targeted the variants c.1117G>T, both homozygous and heterozygous states were clearly distinguishable from wild-type alleles. However, c.1193G>C variant was much more difficult to differentiate due to the formation of a similar number of hydrogen bonds for G>C substitution, and thus similar level bond energy was involved for both the wild-type and mutant variants. To overcome these difficulties, the optimum concentration of MgCl_2_ was determined to be 8 mM for detection of a single G>C point mutation by HRM because 8 mM MgCl_2_ concentration could clearly distinguish among homozygous, heterozygous, and wild-type alleles. This showed that MgCl_2_ could have an effect on HRM studies to differentiate different states of mutation of G/C alleles. For the c.1193G>C variant, the melt curve showed almost similar patterns among the samples with the wild-type allele and also samples with homozygous and heterozygous alleles. However, the temperature-shifted curve could clearly differentiate all the states. Different studies demonstrated that the HRM method could not distinguish purine to pyrimidine nucleotide substitution, such as A to T or G to C substitution, due to the same melting temperature [37]. For the third variant, c.2173A>C, Adenine nucleotide was substituted by Cytosine nucleotide, and due to the difference in bond energy between purine and pyrimidine group, the melting temperature was shifted for both heterozygous and homozygous states compared to the wild-type state. The temperature-shifted pattern was differentiated in such a manner that the wild-type had a lower Tm pattern compared to the heterozygous and homozygous states.

Although Sanger sequencing is the gold standard for mutation detection, HRM can be used as a fast and less expensive approach with 100% sensitivity and specificity for screening and detection of mutations in the *TPO* gene in Bangladeshi patients. Since *TPO* gene variant is inherited in an autosomal recessive manner to cause dyshormonogenesis, this HRM method can also investigate its carrier state.

## 5. Conclusion

High-resolution melt curve analysis could be an effective approach for screening common mutations in the *TPO* gene in Bangladeshi patients with thyroid dyshormonogenesis so that complications of late-diagnosed patients can be prevented by early screening and initiation of treatment in a different strategy.

## Supporting information

S1 Table List of primers used in HRM curve analysis. (DOCX)

S2 Table Reaction setup for HRM protocol. (DOCX)

S3 Table Optimized HRM PCR protocol. (DOCX)

S4 Table Sequenced samples that were used for the HRM method setup. (DOCX)

S5 Table Unknown samples used for HRM method validation. (DOCX)
