# Supplementary material for: High-resolution melt curve analysis: An approach for variant detection in the TPO gene of congenital hypothyroid patients in Bangladesh
Source: PLoS One. 2024 Apr 10;19(4):e0293570. doi: 10.1371/journal.pone.0293570 (PMC11006132; doi:10.1371/journal.pone.0293570)
Supplement: S1 Table — (DOCX) [file pone.0293570.s001.docx]

**High-Resolution Melt Curve Analysis: An Approach for Variant Detection in the *TPO* Gene of Congenital Hypothyroid Patients in Bangladesh**

Mst. Noorjahan Begum^1,2,3^, Rumana Mahtarin^2^, Md Tarikul Islam^2^, Nusrat Jahan Antora^2^, Suprovath Kumar Sarker^,2^, Nusrat Sultana^2^, Abu A. Sajib^1^, Abul B.M.M.K Islam^1^, Hurjahan Banu^4^, M A Hasanat^4^, Kohinoor Jahan Shyamaly^5^, Suraiya Begum^5^, Tasnia Kawsar Konika^6^, Shahinur Haque^6^, Mizanul Hasan^6^, Sadia Sultana^7^, Taufiqur Rahman Bhuiyan^8^, Kaiissar Mannoor^2^, Firdausi Qadri^2,8^, Sharif Akhteruzzaman^1^*

^1^Department of Genetic Engineering & Biotechnology, University of Dhaka, Dhaka-1000, Bangladesh.

^2^Institute for Developing Science and Health Initiatives (ideSHi), ECB Chattar, Mirpur, Dhaka, Bangladesh.

^3^Virology Laboratory, Infectious Diseases Division, International Centre for Diarrhoeal Disease Research, Bangladesh, Mohakhali, Dhaka-1212, Bangladesh.

^4^Department of Endocrinology, Bangabandhu Sheikh Mujib Medical University (BSMMU), Shahbag, Dhaka-1000, Bangladesh.

^5^Department of Pediatrics, Bangabandhu Sheikh Mujib Medical University (BSMMU) Shahbag, Dhaka-1000, Bangladesh.

^6^Nuclear Medicine and Allied Sciences, Bangabandhu Sheikh Mujib Medical University (BSMMU), Shahbag, Dhaka-1000, Bangladesh.

^7^Scintigraphy Division, Bangabandhu Sheikh Mujib Medical University (BSMMU) Shahbag, Dhaka-1000, Bangladesh.

^8^Mucosal Immunology and Vaccinology, Infectious Diseases Division, International Centre for Diarrhoeal Disease Research, Bangladesh, Mohakhali, Dhaka-1212, Bangladesh.

*Correspondence: [sazaman@du.ac.bd](mailto:sazaman@du.ac.bd)

Table S1: List of primers used in HRM curve analysis

| **Primer name** | **Variant**  **(nucleotide position)** | **Primer sequences (5’-3’)** | **Product size (base pair)** |
| --- | --- | --- | --- |
| TPO_G1117T_Ex8 | c.1117G>T | Forward: CGCCTACCTGCCCTTCGTGC | 101 |
|  |  | Reverse: CGTCTCCGGCCAGGAAGCAG |  |
| TPO_G1193C_Ex8 | c.1193G>C | Forward: CTGCTTCCTGGCCGGAGACG | 65 |
|  |  | Reverse: ACAGCGTGTGCAGTGCCGTCAG |  |
| TPO_A2173C_Ex12 | c.2173A>C | Forward: AGACTTTGAGTCTTGTGACAGC | 90 |
|  |  | Reverse: GTGAGAGGAGACCGAACTTCACC |  |
