# Supplementary material for: High-resolution melt curve analysis: An approach for variant detection in the TPO gene of congenital hypothyroid patients in Bangladesh
Source: PLoS One. 2024 Apr 10;19(4):e0293570. doi: 10.1371/journal.pone.0293570 (PMC11006132; doi:10.1371/journal.pone.0293570)
Supplement: S2 Table — (DOCX) [file pone.0293570.s002.docx]

**Table S2. Reaction setup for HRM protocol**

| **Component** | **Volume per 10 µL reation, µL** |
| --- | --- |
| Precision melt super mix (2X) | 5 µL |
| Forward Primer | 0.2 µL |
| Reverse Primer | 0.2 µL |
| MgCl2 | 8mM |
| DNA | 1 µL |
| Nuclease-free water | 3.6 µL |
