# Supplementary material for: High-resolution melt curve analysis: An approach for variant detection in the TPO gene of congenital hypothyroid patients in Bangladesh
Source: PLoS One. 2024 Apr 10;19(4):e0293570. doi: 10.1371/journal.pone.0293570 (PMC11006132; doi:10.1371/journal.pone.0293570)
Supplement: S3 Table — (DOCX) [file pone.0293570.s003.docx]

**Table S3. Optimized HRM PCR protocol**

| **Cycling steps** | **Temperature** | **Time** | **Cycles** |
| --- | --- | --- | --- |
| Initial denaturation | 95°C | 3 min | 1 |
| Denaturation | 95°C | 10s | 40 |
| Annealing | 60°C | 15s |  |
| Extension | 72°C | 15s |  |
| **High Resolution Melting Analysis** | | | |
| Denaturation | 95°C | 30 s | 1 |
| Renaturation | 60°C | 1 min | 1 |
| Melting | 65°C-95°C  (0.1°C-0.2°C increments) | 5s/step | 1 |
