# Supplementary material for: High-resolution melt curve analysis: An approach for variant detection in the TPO gene of congenital hypothyroid patients in Bangladesh
Source: PLoS One. 2024 Apr 10;19(4):e0293570. doi: 10.1371/journal.pone.0293570 (PMC11006132; doi:10.1371/journal.pone.0293570)
Supplement: S4 Table — (DOCX) [file pone.0293570.s004.docx]

**Table S4. Sequenced samples that were used for the HRM method setup**

| **Sl no** | **Specimen** | **Variants in Exon-8** | **Variants in Exon-12** |
| --- | --- | --- | --- |
| 1 | Specimen-01 | c.1117 G→T (p.373Ala→Ser) # c.1193G→C (p.398 Ser→Thr) # | No variant |
| 2 | Specimen-02 | c.1193 G→C (p.398 Ser→Thr) * | c.2173 A→C (p.725 Thr→Pro) # |
| 3 | Specimen-03 | c.1117 G→T (p.373 Ala→Ser)* c.1193 G→C (p.398 Ser→Thr)# | c.2173 A→C (p.725Thr→Pro)* |
| 4 | Specimen-04 | c.1117 G→T (p.373 Ala→Ser)* c.1193 G→C (p.398 Ser→Thr)* | c.2173 A→C (p.725Thr→Pro)* |
| 5 | Specimen-05 | c.1193 G→C (p.398 Ser→Thr) # | c.2173 A→C (p.725Thr→Pro)* |
| 6 | Specimen-06 | c.1117 G→T (p.373 Ala→Ser)* c.1193 G→C (p.398 Ser→Thr)* | No variant |
| 7 | Specimen-07 | c.1117 G→T (p.373 Ala→Ser)* c.1193 G→C (p.398 Ser→Thr) # | No variant |
| 8 | Specimen-08 | c.1117 G→T (p.373 Ala→Ser)* c.1193 G→C (p.398 Ser→Thr) # | c.2173 A→C (p.725Thr→Pro)* |
| 9 | Specimen-09 | No variant | c.2173 A→C (p.725 Thr→Pro) # |
| 10 | Specimen-10 | c.1193 G→C (p.398 Ser→Thr) # | c.2173 A→C (p.725 Thr→Pro) # |
| 11 | Specimen-11 | No variant | c.2173 A→C (p.725 Thr→Pro) # |
| 12 | Specimen-12 | c.1117 G→T (p.373 Ala→Ser)* c.1193 G→C (p.398 Ser→Thr)* | c.2173 A→C (p.725 Thr→Pro)* |
| 13 | Specimen-13 | c.1193 G→C (p.398 Ser→Thr) * | c.2173 A→C (p.725 Thr→Pro) # |
| 14 | Specimen-14 | c.1117 G→T (p.373 Ala→Ser)* c.1193 G→C (p.398 Ser→Thr)* | c.2173 A→C (p.725 Thr→Pro)* |
| 15 | Specimen-15 | c.1117 G→T (p.373 Ala→Ser)* c.1193 G→C (p.398 Ser→Thr) # | No variant |
| 16 | Specimen-16 | c.1193 G→C (p.398 Ser→Thr) * | c.2173 A→C (p.725 Thr→Pro)* |
| 17 | Specimen-17 | c.1117 G→T (p.373 Ala→Ser) # c.1193 G→C (p.398 Ser→Thr) # | No variant |
| 18 | Specimen-18 | c.1117 G→T (p.373 Ala→Ser)* c.1193 G→C (p.398 Ser→Thr) # | No variant |
| 19 | Specimen-19 | c.1117 G→T (p.373 Ala→Ser)* c.1193 G→C (p.398 Ser→Thr) # | No variant |
| 20 | Specimen-20 | c.1117 G→T (p.373 Ala→Ser) # c.1193 G→C (p.398 Ser→Thr) # | No variant |
